# Encapsulated fat necrosis mimicking abdominal liposarcoma: A case report and literature review

**DOI:** 10.1002/ccr3.3120

**Published:** 2020-07-12

**Authors:** Jun Watanabe, Tomohiro Osaki, Shigeru Tatebe, Keisuke Goto, Kanenori Endo, Seiichi Nakamura, Yasuaki Hirooka

**Affiliations:** ^1^ Department of Surgery Tottori Prefectural Central Hospital Tottori Japan

**Keywords:** abdominal, fat necrosis, liposarcoma, surgery

## Abstract

We report a case of an encapsulated fat necrosis without significant medical history. To differentiate from liposarcoma, it should be recognized that a half of abdominal encapsulated fat necrosis cases have a history of inflammation and surgery.

## Graphical abstract

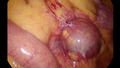

## 1 INTRODUCTION

Encapsulated fat necrosis is a rare inflammatory disorder of fat tissue, presenting as an intra‐abdominal mass after pancreatitis, trauma, or abdominal surgery.^1^ Encapsulated fat necrosis was first reported in the breast in 1978.^2^ It is most likely to occur in the extremities such as the feet, knees, thighs, arms, the hip, chest, and finally the abdomen.^3^ In addition, fat necrosis is benign and usually asymptomatic and therefore may be difficult to differentiate from liposarcoma using the clinical history and imaging alone.^4^ Although the clinical features of encapsulated fat necrosis in subcutaneous lesions are well described, its features in the abdomen have not fully been summarized to date.^3^

Herein, we report a case of laparoscopic resection of an encapsulated fat necrosis without significant medical history other than ureterolithiasis and review the relevant literature.

## 2 CASE PRESENTATION

**FIGURE 1:**
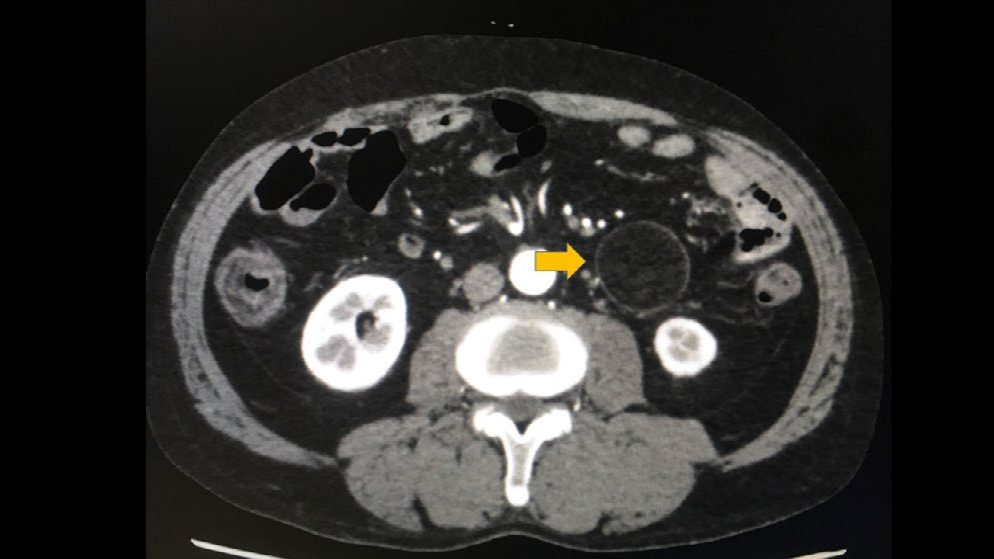
Computed tomographic image of the abdomen, demonstrating a round and encapsulated 4 × 4 cm mass without any contrast enhancement

**FIGURE 2:**
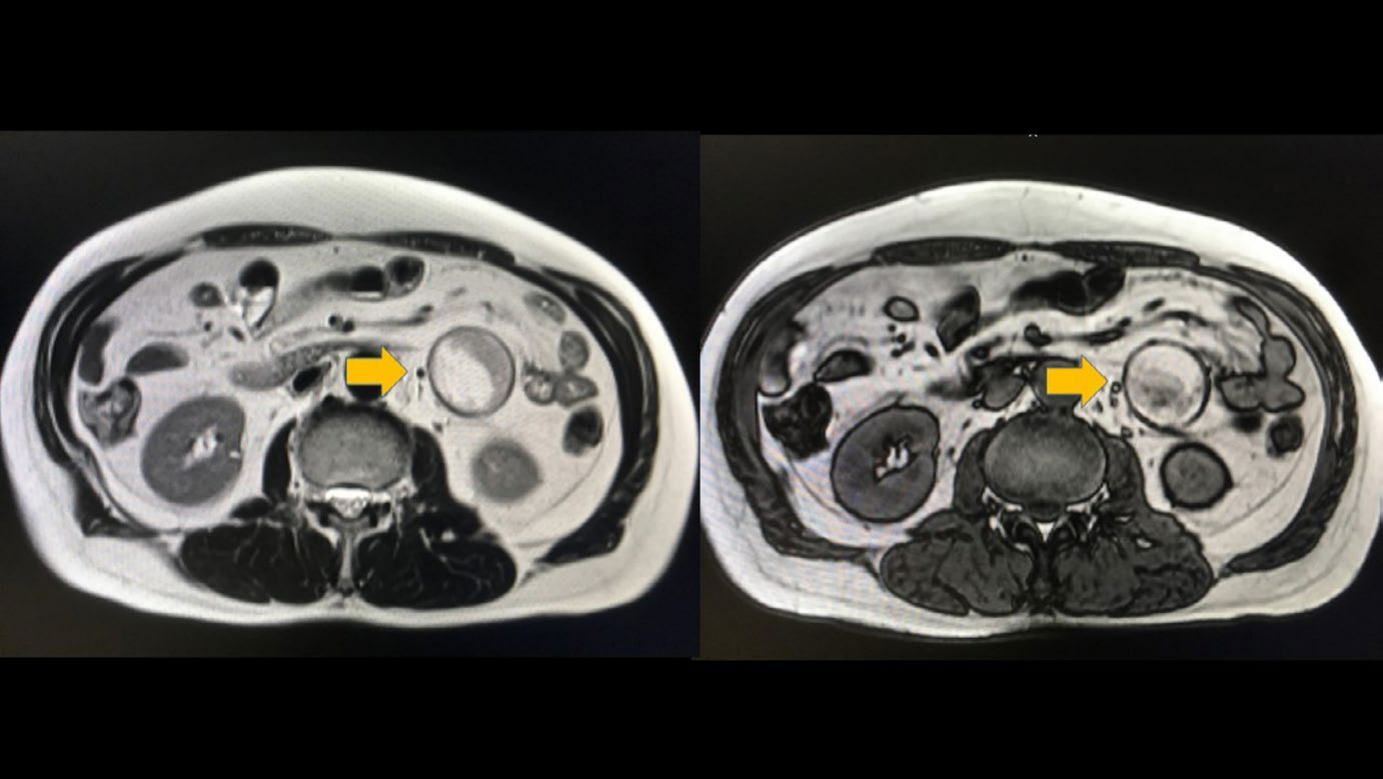
Left: Magnetic resonance imaging T1W and Right: Magnetic resonance imaging T2W showed a round lesion with a thick hypointense wall and a central fatty focus

**FIGURE 3:**
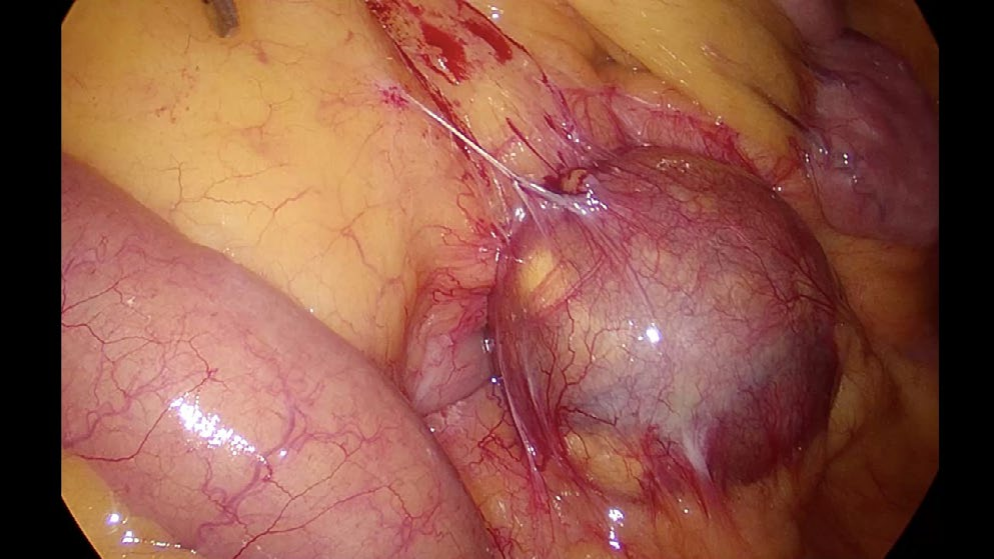
Laparoscopic image of the tumor on the descending mesocolon

**FIGURE 4:**
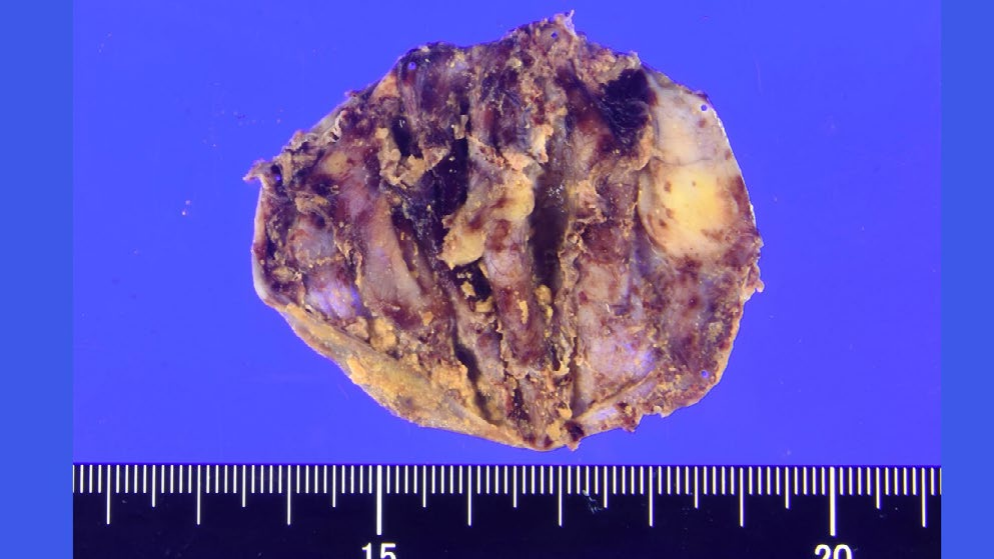
Macroscopic findings

**FIGURE 5:**
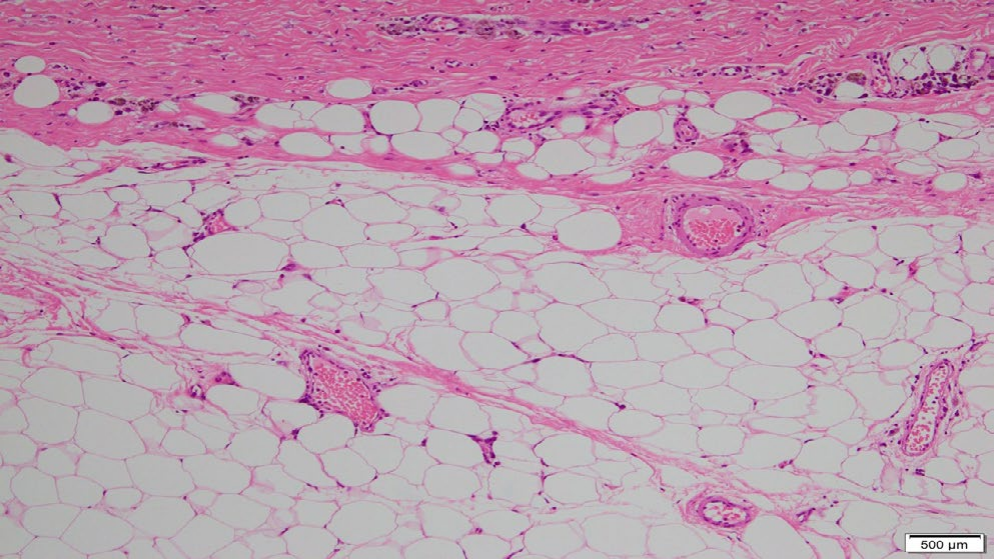
Histopathological findings

A 69‐year‐old man was referred to our hospital for evaluation of a painless abdominal mass within his left abdomen that was incidentally discovered during assessment for ureterolithiasis. However, according to the patient's medical documents, there was no abdominal mass 4 years ago on computed tomography (CT) scan. He had no history of pancreatitis, trauma, or abdominal surgery. The physical examination did not reveal any abdominal tenderness or a palpable mass. Laboratory tests were normal, including hepatobiliary function, renal function, inflammatory response, and urinalysis. CT scan showed a round and encapsulated 4 × 4 cm mass without any contrast enhancement in the upper left abdomen (Figure 1). There was no evidence of lymphadenopathy or distant metastasis of liver and lungs. Magnetic resonance imaging showed that the tumor on the descending mesocolon had high‐intensity (SI) portions on T1‐weighted images (WIs) and iso‐intense portions on T2‐WIs compared to the fat. Comparison of iso‐SI to the fat showed fat suppression on T2‐weighted short tau inversion recovery images. There was a thin rim at the periphery of the mass showing low SI on both T1‐ and T2‐WIs and revealing contrast enhancement (Figure 2). Imaging findings could not positively suspect malignant signs, but a 4‐cm‐sized tumor that suddenly occurred during the last 4 years with no history of pancreatitis, trauma, or abdominal surgery could not completely rule out liposarcoma. Close follow‐up was also offered to patients as an alternative to surgeries; however, liposarcoma was undeniable and the surgery was conducted for definitive diagnosis and treatment. The tumor on the descending mesocolon was removed laparoscopically (Figure 3). The adhesion around the tumor was peeled and removed without breaking the capsule. There were no adhesion and inflammation in the appendix, appendices epiploicae, omentum, and retroperitoneum around the kidney and ureter. Histopathological findings revealed that the tumor wall comprised hyalinized fibrous connective tissue and the inside of the tumor consisted of degenerated and necrotic fatty tissue of the same size; it was diagnosed as encapsulated fat necrosis (Figures 4 and 5). The patient's postoperative course was uneventful, and he was discharged 5 days later. Presently, about 1 year after surgery, the patient remains healthy and has no signs of recurrence or complications.

## 3 DISCUSSION

Encapsulated fat necrosis can exist as an intra‐abdominal mass without pancreatitis, trauma, or abdominal surgery. Laparoscopic resection of encapsulated fat necrosis is acceptable if liposarcoma cannot be ruled out by clinical history and imaging findings.

Although encapsulated fat necrosis usually occurs after pancreatitis, trauma, or abdominal surgery, the etiology in the patient described is unclear. A PubMed database search was performed using the terms “encapsulated” OR “nodular” AND “fat necrosis” up to February 2020 and nine articles were retrieved.^5^, ^6^, ^7^, ^8^, ^9^, ^10^, ^11^, ^12^, ^13^ The citation lists of the extracted articles were searched through Google Scholar for further identification of additional studies. Table 1 summarizes the characteristics of previous nine patients (seven males and two females) diagnosed with abdominal encapsulated fat necrosis with the average age of 54 years (range, 20‐78 years). Eight patients had some symptoms, four indicating with abdominal pain, and one was incidentally found on CT scan. Two patients had a history of appendectomy, two had appendicitis or colonic diverticulosis, and five had no history of surgery or injury. Most patients underwent laparoscopic resection. The literature review revealed that the encapsulated fat necrosis may occur in traumatic injury and abdominal surgery, but the cause may not be well understood in most cases.

**TABLE 1:** Summary of the characteristics of abdominal encapsulated fat necrosis

Author (ref)/y	Age	Sex	Chief complaint	Cause	Location	Size	Treatment
Felipo et al^5^/2004	67	Male	Morbid obesity	Appendectomy	Intraperitoneal	14.0 cm	Surgery
Unal et al^6^/2005	20	Male	Abdominal pain	Appendectomy	Intraperitoneal	2.0 cm	Surgery
Gupta et al^7^/2007	45	Female	Menorrhagia	NR	Ovary	NR	Surgery
Lee et al^8^/2010	60	Male	Abdominal pain	Without injury	Retroperitoneum	33.0 cm	Laparotomy
Brooke and Choti^9^/2011	56	Male	Chest pain	NR	Intraperitoneal	3.2 cm	Laparotomy
Oh et al^10^/2015	66	Male	None	Appendicitis	Intraperitoneal	NR	Laparotomy
De Kock and Delrue^11^/2016	33	Female	Abdominal pain	NR	Intraperitoneal	NR	NR
Magalhães et al^12^/2016	78	Male	Abdominal pain	Diverticulitis	Intraperitoneal	3.8 cm	Laparotomy
Biondi et al^13^/2017	59	Male	Diarrhea and fever	Without surgery	Intraperitoneal	6.5 cm	Laparotomy

Abbreviation: NR, not reported.

Laparoscopic resection of encapsulated fat necrosis is acceptable if there are no signs of pancreatitis, trauma, and abdominal surgery, and imaging findings do not rule out liposarcoma. Although encapsulated fat necrosis can be monitored as an alternative to resection due to its nature of shrinking in size over the time, pathological analysis with surgical resection is critical in making the final diagnosis.^14^

Because the natural progression is to decrease in size over time, pathological analysis with surgical resection is critical in making the final diagnosis.^14^ Septum enhancement and/or thick septa of 2 mm or more in the intra‐abdominal cystic masses containing fat are presumed liposarcomas; however, in this case, the absence of a septum within the mass was difficult to distinguish from liposarcoma.^14^ On our radiological findings, well‐differentiated liposarcoma, spindle cell lipoma, and fat necrosis were diagnosed because the tumor mainly consisted of both fat and nonlipomatous components.^15^ Macroscopically, liposarcoma is generally well circumscribed but not encapsulated, which may depend on tumor differentiation.^16^ Although spindle cell lipoma is more suggestive when the tumor occurs in middle‐aged men, especially localized behind the neck, in this case, the tumor was located in the abdominal cavity of older men.^17^ The deep typical lesions, such as retroperitoneum and deep thigh musculature, of liposarcoma require biopsy for definitive diagnosis.^18^ However, in the abdominal cavity, the liposarcoma is at risk of intraperitoneal dissemination, so it is more important to remove the tumor without breaking the capsule than to biopsy the mass. In the histopathological findings, liposarcoma showed adipocytes of different sizes and atypical stromal cells in the fibrous septum, but in the present case, the above findings, including lipoblast, were not observed.^16^ Therefore, additional immunohistochemical study was not added.

In conclusion, encapsulated fat necrosis can present as an abdominal mass with no significant history and laparoscopic resection is indicated. We should be aware that half of the encapsulated fat necrosis mimicking liposarcoma can manifest as an intra‐abdominal mass with histories of pancreatitis, trauma, and abdominal surgery. Encapsulated fat necrosis with a well‐defined cause may allow for careful follow‐up.

## CONFLICT OF INTEREST

None declared.

## AUTHOR CONTRIBUTION

JW and KE: conceived and designed the study. KG, TO, and TS: revised the manuscript. SN and YH: were involved in overall supervision of the paper. All authors read and approved the final manuscript.

## ETHICS APPROVAL AND CONSENT TO PARTICIPATE

Ethics approval was not necessary for this case report. All patient's data and photographs are de‐identified.

## INFORMED CONSENT

Written informed consent was obtained from the patient for publication of this case report and any accompanying images.

## Data Availability

Data sharing is not applicable to this article as no datasets were generated or analyzed during the current study.
